# History, ECG, Risk Factors (HER) Scoring for Cardiac Risk Stratification in Patients <45 Years of Age Presenting With Chest Pain

**DOI:** 10.7759/cureus.40458

**Published:** 2023-06-15

**Authors:** Christopher A Legare, Erica Dunn, Kate Arner, Kylie Ridley, Tristan Diaz, Holly Stankewicz, Rebecca Jeanmonod

**Affiliations:** 1 Emergency Medicine, St. Luke’s University Health Network, Bethlehem, USA; 2 Emergency Medicine, Lewis Katz School of Medicine at Temple University, Bethlehem, USA

**Keywords:** common emergency department complaints, young patients, troponin, mace, risk stratification, chest pain

## Abstract

Background

Chest pain is a common chief complaint of patients presenting to the emergency department. Acute coronary syndrome (ACS) is found to be the etiology of this symptom in a minority of these patient encounters. This study aimed to determine the utility of using the History, ECG, Risk Factors (HER) components of the History, ECG, Age, Risk Factors, Troponin (HEART) score in ruling out 30-day Major Adverse Cardiac Event (MACE), ACS, ventricular tachycardia, and ventricular fibrillation in patients aged less than 45. Additionally, the utility of this score in ruling out a positive troponin was investigated as well.

Methodology

This is a retrospective chart review study that examined a consecutive cohort of 7,724 patients presenting with chest pain to the 11 emergency departments of a single healthcare system over a two-year period (January 2019 to December 2020). HER scores of 0 to 1 were categorized as negative (-) and scores of two or greater were categorized as positive (+). Sensitivity, specificity, and predictive values were calculated for the relationship between HER score positivity and primary cardiac disease and troponin results.

Results

Test characteristics of HER scoring for significant primary cardiac disease in patients between 18 and 45 years of age presenting with undifferentiated chest pain were sensitivity of 88.0 (CI = 80.0-94.0), specificity of 72.6 (CI = 71.8-73.8), positive predictive value of 3.1 (CI = 2.4-3.9), and negative predictive value of 99.8 (CI = 99.7-99.9). Furthermore, an HER score >1 was neither sensitive nor specific in predicting a positive troponin (sensitivity = 80, CI = 71.9-86; specificity = 71.3, CI = 70.3-72.3). However, the negative predictive value of an HER score of 0-1 was 99.5 (CI = 99.3-99.7) and the positive predictive value was 4.7 (CI = 3.9-5.7).

Conclusions

According to this study, when evaluating young patients who are deemed to have a subjectively non-highly suspicious history, who have minimal risk factors, and who have an ECG without significant ST deviation, troponin testing is low yield in the risk stratification of patients under the age of 45 for serious primary cardiac disease.

## Introduction

Ischemic heart disease is a leading cause of death among adults in the United States. Approximately 90% of people <56 years old diagnosed with acute myocardial ischemia (AMI) (ST-elevation myocardial infarction (STEMI), non-ST-elevation myocardial infarction (NSTEMI), unstable angina) present with chest pain [1]. Unfortunately, chest pain is one of the most common complaints in the emergency department (ED), accounting for more than 5% of all ED visits [2]. Ultimately, only 13% of these are due to acute coronary syndrome (ACS) [3]. The History, ECG, Age, Risk Factors, Troponin (HEART) score, which assigns up to two points to each component that are summed to a total correlating with the risk of Major Adverse Cardiac Event (MACE), is a validated risk stratification tool for this patient population; however, to use this tool, bloodwork for troponin testing is required and a repeat troponin is commonly necessary for physicians to decide whether or not to admit the patient [4-6]. This adds time to ED patient disposition and costs to the healthcare system [7,8]. Given the negative association between ED crowding and adverse clinical outcomes, we must evaluate ways to reduce excess testing and decrease disposition time [9,10]. Furthermore, ED length of stay is inversely associated with patient satisfaction [11].

The primary endpoint of this study was to determine the utility of using the History, ECG, Risk Factors (HER) components of the HEART score in ruling out 30-day MACE, ventricular tachycardia, and ventricular fibrillation in patients aged less than 45 years. As described in the HEART score derivation study, MACE was defined as a composite outcome of AMI, percutaneous coronary intervention (PCI), coronary artery bypass graft (CABG), and death [4]. ACS was also considered to be a component of MACE in this study. Additionally, given the vulnerability of MACE to workup bias and how troponin testing is a commonly used objective endpoint for disposition planning in ED clinical practice, we investigated the predictive potential of HER scoring for troponin positivity [6].

This article was previously presented as an abstract poster presentation at the 2023 American Academy of Emergency Medicine (AAEM) Scientific Assembly on April 24th, 2023, and as a meeting abstract at the 2023 Pennsylvania College of Emergency Physicians (PACEP) Scientific Assembly on May 5th, 2023.

## Materials and methods

Study design

This is a retrospective chart review study that examined a consecutive cohort of patients less than 45 years of age presenting to a single health network with a chief complaint of chest pain. The study protocol was reviewed by the network institutional review board and found to be exempt.

Study setting and population

The study was performed at a single health network that includes 11 EDs. The EDs of this health network, located in the Mid-Atlantic region of the United States, encompass a variety of settings, including urban, suburban, and rural. Six of these hospitals are accredited chest pain centers and four are PCI sites. Annual network volumes, across all 11 EDs, total over 320,000 patient encounters. All EDs are staffed by providers that include board-certified/eligible emergency physicians as well as, depending upon the location, physician assistants and emergency medicine residents. Notably, as it is a quality metric component of the chest pain center accreditation process, ED provider documentation of HEART scores for all patient encounters with an ED diagnosis of chest pain is expected and tracked by the health network. The catchment area of the study health network is shared by a second large health network that utilizes the same electronic medical record (EMR), allowing for a review of any patient laboratory values or encounter information during the study follow-up period.

All patients less than 45 years of age presenting to one of our health network’s EDs with a chief complaint of chest pain and who were risk stratified with the HEART score were eligible for inclusion in this study. Patients who left without being seen by a provider, those without documentation of the HEART score, and those less than 18 years of age were excluded. The study period was two years from January 1st, 2019, to December 31st, 2020.

Study protocol and measurements

All patient encounters were electronically extracted from the health-network-wide EMR (EPIC Systems, Verona, WI, USA) by a primary chief complaint of chest pain. Chief complaints within our healthcare system are placed in the chart by registration personnel or by nursing staff during the triage process. Additional data including patient age, sex, troponin, HEART score, final diagnosis, and disposition were automatically populated from the EMR into a standardized Excel (Microsoft Corporation, Redmond, WA, USA) spreadsheet through the chart abstraction tool. Each chart was then manually reviewed by trained research associates to determine which individual HEART score elements contributed to the overall score recorded in the chart. If multiple HEART scores were recorded, the latest timestamped value was used. The HER score was calculated from this data. For the purposes of this study, HER scores of 0-1 were categorized as negative (-), and scores of 2 or greater were categorized as positive (+). An HER score of 0-1 was chosen as negative for several reasons. First, by definition, there is a minimal subjective perceived risk from the provider. Second, cardiac risk factors in younger adults are associated with cardiovascular pathology in older adults but this is not as established for younger adults [12]. Finally, non-specific ST-T changes on ECG are found in a sizable proportion of the population [13]. Therefore, there is minimal objective risk in the young study population with HER scores of 0-1 as well.

Research associates also determined whether patients underwent cardiology consultation, cardiac stress testing, catheterization, or had further healthcare contact within the subsequent six-week follow-up period and recorded these results in the spreadsheet. ECG review was performed for patients with a diagnosis of arrhythmia, myocardial infarction (MI), bypass, or PCI to determine if STEMI was present. These reviews encompassed both our own healthcare network as well as the region’s other large healthcare network. Ultimately, data included HEART scores, HER scores with relevant components, demographics, disposition, 30-day-MACE, 30-day mortality, troponin positivity, catheterization results, and final diagnosis. Missing data, regarding documented HER/HEART score components in the medical record, were assumed to be 0 during data analysis. This was done as missing data does not add to the calculated total score in the EMR and the total score is presumably what was used during clinical decision-making.

Investigator training

All research associates were provided with written step-by-step instructions outlining how to search the medical record and abstract data from each patient encounter. Each research associate was provided with a consecutive block of patient encounters for which they were responsible. A random selection of patient encounters from each block was reviewed by the primary investigator (PI) for the accuracy of HER scoring. All patient encounters in which the HER score did not equal the HEART score (in other words, patients who received points for troponin as no patients in our cohort received points for age) were also reviewed by the PI. In the infrequent case where there was disagreement between reviewers, the PI made the ultimate decision on data entry before analysis. The PI was not involved in data analysis.

Data handling and analysis

Data were analyzed using descriptive statistics and chi-square using MedCalc (1993-2013, Ostend, Belgium) and Microsoft Excel 2007 (Microsoft Corporation, Redmond, WA, USA). Patient demographic and HER score data were non-parametric and reported as medians with quartiles and descriptive statistics, respectively.

## Results

Study population demographics

**Figure 1:**
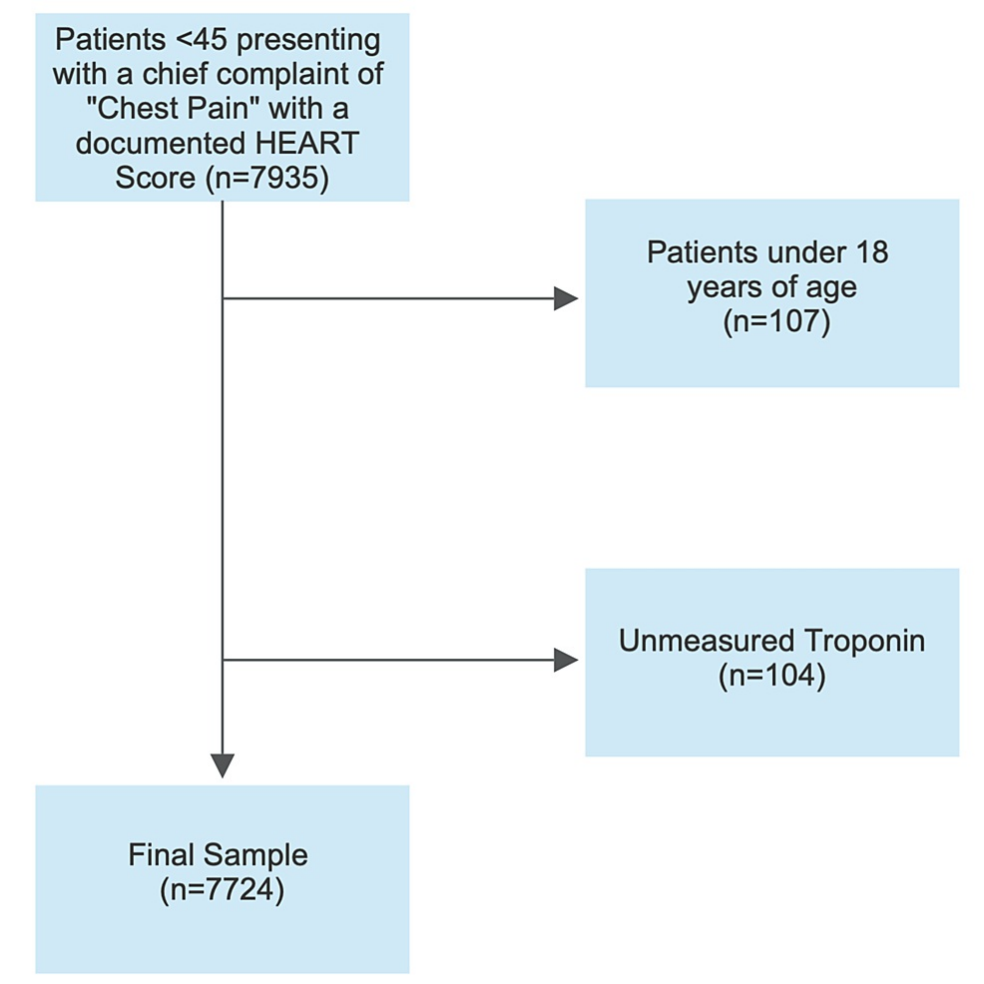
Study inclusion and exclusion.

A total of 7935 patients under the age of 45 presented to the study EDs with a chief complaint of chest pain and were enrolled in the study. In total, 107 were excluded from the analysis as they were under the age of 18. Furthermore, despite a documented HEART score, an additional 104 were not included because troponin was not tested, leaving 7,724 for analysis (Figure 1).

**Figure 2:**
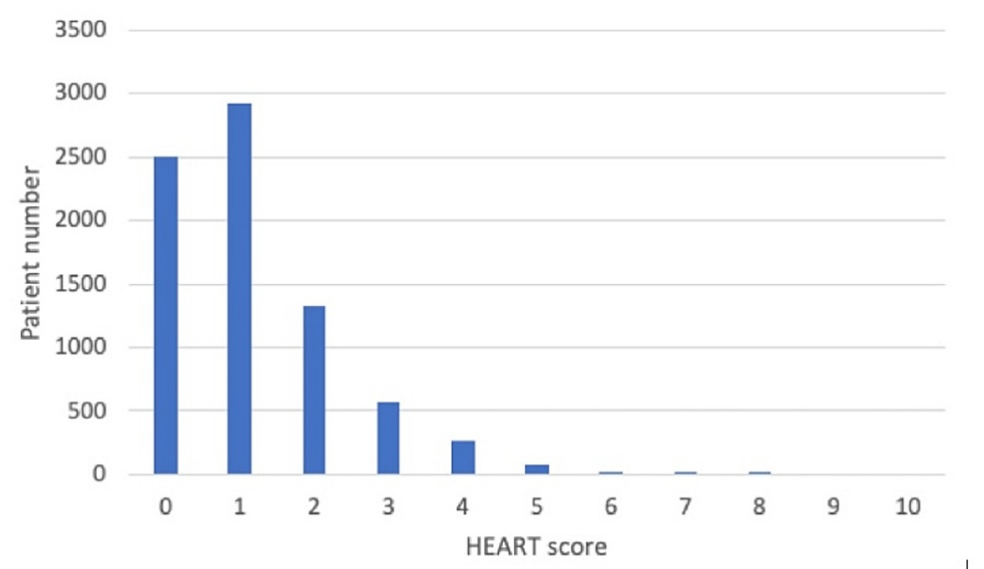
Distribution of HEART scores.

The population was 47.4% male and 52.6% female. The remainder of the demographic information is demonstrated in Table 1. HEART scores ranged from 0 to 8 and are shown in Figure 2. HER scores ranged from 0 to 6. Men had median HER scores of 1 (IQR = 0-2), and women had median HER scores of 1 (IQR = 0-1, p < 0.0001). Most patients (87.2%) were discharged, and the disposition of the remainder is listed in Table 1. The correlation between age and HEART score was weak with an R^2^ of 0.1.

**Table 1:** Baseline characteristics.

	Total (n = 7,724)
Median age (IQR)	35 (29–40)
Gender (%)	
Male	3,661 (47.4)
Female	4,063 (52.6)
Self-identified race (%)	
White	4,959 (64.2)
Black	1,081 (14)
Hispanic	147 (1.9)
Multiracial or other	1,306 (16.9)
Not documented	231 (3)
Disposition (%)	
Discharged	6,735 (87.2)
Admitted	765 (9.9)
Direct to the cath lab	6 (0.08)
Died	1 (0.01)
Transfer to behavioral or labor and delivery	217 (2.8)

Primary endpoint

**Figure 3:**
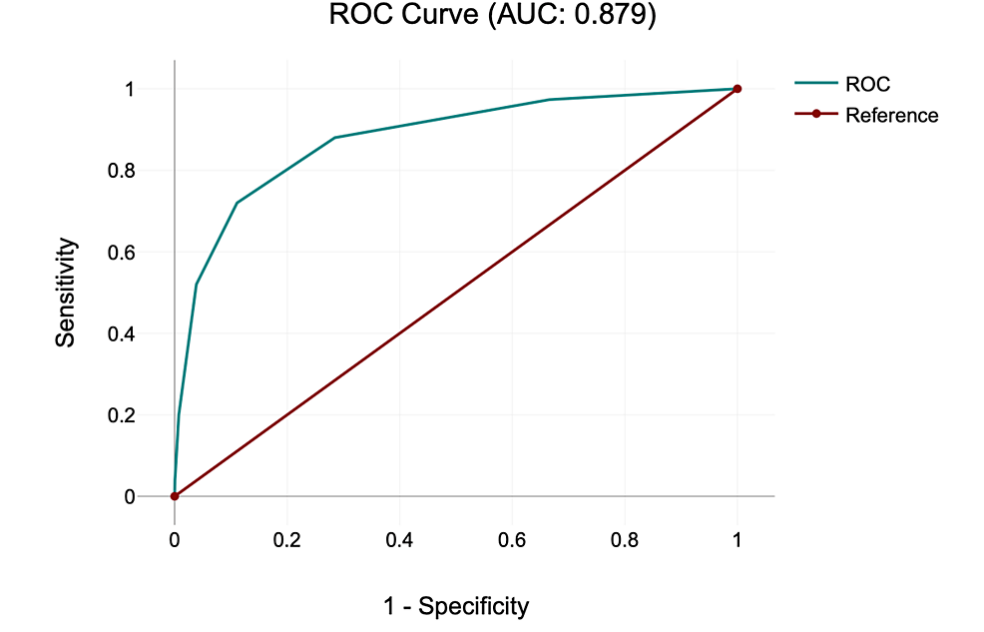
Receiver operating curve for the HER score and serious primary cardiac diagnosis.

About 1% (n = 75, 0.96%, CI = 0.76-1.2) of patients between the ages of 18 and 45 presenting to the ED with a chief complaint of chest pain were found to have a significant primary cardiac diagnosis (30-day MACE, ventricular tachycardia, and ventricular fibrillation). Of these, 88% (n = 66) had an HER score >1, and 12% (n = 9) had an HER score of 0 or 1. Test characteristics of HER scoring for significant primary cardiac diagnosis in patients between 18 and 45 years of age presenting with undifferentiated chest pain were sensitivity of 88.0 (CI = 80.0-94.0), specificity of 72.6 (CI = 71.8-73.8), positive predictive value of 3.1 (CI = 2.4-3.9), and negative predictive value of 99.8 (CI = 99.7-99.9) (Table 2). Additionally, a receiver operating curve was created for the HER score and serious primary cardiac diagnosis (Figure 3). The area under the curve was 0.879 showing, along with the sensitivity, that this test is discriminatory.

**Table 2:** Primary outcome test characteristics.

Parameter	
Prevalence	1.0%
Sensitivity	88.5% (78.7–94.3)
Specificity	73.5 (78.7–94.3)
Positive predictive value	3.3 (2.6–4.2)
Negative predictive value	99.8 (99.7–99.9)

For patients with an HER score >1, nine had STEMI noted on the ECG, 30 had highly suspicious stories, and 42 had three or more risk factors. None of the patients who were discharged, transferred to other services, or left against medical advice were found to have mortality or MACE at 30 days. About 11% of admitted patients underwent cardiac catheterization (n = 87). Of these, 5 were diagnosed with cardiac disease. These five patients had HER scores >1.

Secondary endpoint

The prevalence of a positive initial troponin, determined to be positive by a normal/elevated cutoff that is defined by the institution/laboratory and applied uniformly throughout the network, in this patient population was 1.7% (IQR = 1.5-2.1). An HER score >1 was neither sensitive nor specific in predicting a positive troponin (sensitivity = 80, CI = 71.9-86; specificity = 71.3, CI = 70.3-72.3). That said, due to the incredibly low positive rate of troponin testing in this population, the negative predictive value of an HER score of 0-1 was 99.5 (CI = 99.3-99.7) and the positive predictive value was 4.7 (CI = 3.9-5.7) (Table 3). Ultimately, more patients with positive troponin testing had non-ACS-related diseases, such as myocarditis or pulmonary embolism, as determined by further medical testing and/or cardiologist consultation.

**Table 3:** Secondary outcome test characteristics.

Parameter	
Prevalence	1.7%
Sensitivity	80.0% (71.9–86.0)
Specificity	71.3 (70.3–72.3)
Positive predictive value	4.7 (3.9–5.7)
Negative predictive value	99.5 (99.3–99.7)

## Discussion

The American College of Emergency Physicians published a clinical policy in 2018 stating that a 1-2% miss rate of 30-day MACE diagnosis in non-ST-elevation ACS is acceptable [14]. In the study population of patients between 18 and 44 years of age presenting with undifferentiated chest pain, the risk of serious primary cardiac disease, the composite outcome used in this study that includes 30-day MACE, is about 1%. Therefore, even with no testing at all, the prevalence of 30-day MACE in this population is already within the level of tolerability. Risk stratification through HER scoring, with its sensitivity of 88% and >99% negative predictive value when applied to this population, appears to be a useful and safe tool to further narrow down who might benefit from workup beyond history and an ECG. Stated another way, this study suggests, when evaluating young patients deemed to have a history of present illness that is felt to be non-suspicious for AMI by the healthcare provider, who have minimal risk factors, or who have an ECG without significant ST deviation, that troponin testing is low yield in the risk stratification of patients under the age of 45 for serious primary cardiac disease. Additionally, although not directly analyzed in this study, implementation of this strategy may have the further benefits of improving disposition times, resource allocation, patient satisfaction, and clinical outcomes for the reasons described in the introduction.

Furthermore, for patients with major cardiac-related diagnoses, the greatest contributors to the HER score were history of present illness and past medical history. While ECG is clearly a helpful and necessary part of the diagnostic criteria for STEMI, these data lend support to subjective criteria and physician gestalt for the risk stratification of otherwise healthy young patients presenting to EDs with chest pain.

Despite the implications of these findings in regards to troponin testing in the ED, it is important to consider that, as more patients with positive troponins had non-ACS disease, this test likely has a role in the diagnosis and risk stratification of other disease processes. Most commonly, these non-ACS troponin elevations were secondary to myocarditis and pulmonary embolism. It is reasonable, in patients in whom history is suggestive of either of these disease processes, despite being of a young age and without primary cardiac risk factors, to continue to measure troponin during their evaluation.

Our study suggests there are opportunities for future and further research that evaluates patient characteristics and clinically important outcome differences between those who present with a chief complaint of chest pain and ultimately end up with a non-cardiac disease or chest pain-related diagnosis, therefore purposely eliminating the need for troponin testing and HEART pathway management, versus those who are dispositioned with either chest pain or cardiac disease-related diagnosis. Additionally, as the history and risk factor components of HER scoring seem to have the strongest predictive potential for serious primary cardiac disease in this population, it would be helpful to further delineate which specific historical and risk factors are most responsible for increasing this risk. Further study could also investigate the hypothesis of the current study in a prospective manner, thus increasing the strength of the findings.

Limitations

There are a few notable limitations of this study. Primarily, as the study is retrospective in design, it is subject to the accuracy and completeness of the information placed in the chart. Workup bias is another limitation of this research. When considering the prevalence of MACE in this study population, it is important to note that only about 1% of patients underwent the gold-standard diagnostic test of cardiac catheterization. In a similar way, it is left to the discretion of the treating provider which patients presenting with chest pain have troponin testing performed and HEART score risk stratification applied. As described in the methods section of this study, we only included patients who had a documented HEART score. Therefore, it is important to consider that a HEART score is only recorded 63.2% of the time for all patients presenting with chest pain within our health network and that younger age is an independent predictor of failure to perform risk stratification [15]. While this suggests that, in effect, HER scoring or a gestalt-based variant of this is already being applied in clinical practice to some extent, this limits the breadth of the patients enrolled in this study.

It is also important to reflect on the limitation of missing data. As stated above, 104 patients with a documented HEART score did not have troponin testing during that ED encounter. Furthermore, of those patients with a diagnosis relevant to the study (30-day MACE, Vtach, etc.), 16 were missing at least one HEART score variable in the EPIC tool, i.e., click boxes were completed for several HEART variables but others were left blank causing them to be populated as 0. Only two patients in this subset, with an HER score of 0 or 1, had all of the HEART score variables populated. Overall, this limitation causes the results of this study to be a conservative estimate of the “true” rate of a “negative” HER score with positive results.

Finally, our study is at a single health network using a specific EMR and may not be generalizable to other health networks in other regions.

## Conclusions

Overall, this study shows that, in very low-risk patients with chest pain, history and ECG are sufficient to exclude symptomatic coronary disease and 30-day MACE. Stated another way, troponin testing is low yield in this patient population when investigating for serious primary cardiac disease. It is important to note, however, that troponin testing likely has a role in the diagnosis and risk stratification of other disease processes as more patients with positive troponins had non-ACS disease. Lastly, for patients with major cardiac-related diagnoses, the greatest contributors to the HER score were history of present illness and past medical history.
